# Performance of ethnic minority versus White doctors in the MRCGP assessment 2016–2021: a cross-sectional study

**DOI:** 10.3399/BJGP.2022.0474

**Published:** 2023-03-21

**Authors:** Aloysius Niroshan Siriwardena, Vanessa Botan, Nicki Williams, Kim Emerson, Fiona Kameen, Lindsey Pope, Adrian Freeman, Graham Law

**Affiliations:** Community and Health Research Unit, University of Lincoln, Lincoln; research and development lead for assessment, Royal College of General Practitioners, London.; Community and Health Research Unit, University of Lincoln, Lincoln.; Health Education England, London.; Royal College of General Practitioners, London.; Royal College of General Practitioners, London.; School of Medicine, Dentistry and Nursing, University of Glasgow, Glasgow.; Medical School, University of Exeter, Exeter.; Community and Health Research Unit, University of Lincoln, Lincoln.

**Keywords:** differential attainment, education, medical, ethnic and racial minorities, general practice, medical licensing

## Abstract

**Background:**

Differential attainment has previously been suggested as being due to subjective bias because of racial discrimination in clinical skills assessments.

**Aim:**

To investigate differential attainment in all UK general practice licensing tests comparing ethnic minority with White doctors.

**Design and setting:**

Observational study of doctors in GP specialty training in the UK.

**Method:**

Data were analysed from doctors’ selection in 2016 to the end of GP training, linking selection, licensing, and demographic data to develop multivariable logistic regression models. Predictors of pass rates were identified for each assessment.

**Results:**

A total of 3429 doctors entering GP specialty training in 2016 were included, with doctors of different sex (female 63.81% versus male 36.19%), ethnic group (White British 53.95%, minority ethnic 43.04%, and mixed 3.01%), country of primary medical qualification (UK 76.76% versus non-UK 23.24%), and declared disability (disability declared 11.98% versus not declared 88.02%). Multi-Specialty Recruitment Assessment (MSRA) scores were highly predictive for GP training end-point assessments, including the Applied Knowledge Test (AKT), Clinical Skills Assessment (CSA), Recorded Consultation Assessment (RCA), and Workplace-Based Assessment (WPBA) and Annual Review of Competency Progression (ARCP). Ethnic minority doctors did significantly better compared with White British doctors in the AKT (odds ratio [OR] 2.05, 95% confidence interval [CI] = 1.03 to 4.10, *P* = 0.042). There were no significant differences on other assessments: CSA (OR 0.72, 95% CI = 0.43 to 1.20, *P* = 0.201), RCA (OR 0.48, 95% CI = 0.18 to 1.32, *P* = 0.156), or WPBA—ARCP (OR 0.70, 95% CI = 0.49 to 1.01, *P* = 0.057).

**Conclusion:**

Ethnic background did not reduce the chance of passing GP licensing tests once sex, place of primary medical qualification, declared disability, and MSRA scores were accounted for.

## INTRODUCTION

The role of doctors’ ethnic group in differential attainment in the UK Membership of the Royal College of General Practitioners (MRCGP) licensing assessments is a continuing concern,^1^ and causes are poorly understood.^2^ A study by Esmail and Roberts suggested, despite lack of supportive evidence, that *‘subjective bias due to racial discrimination in the clinical skills assessment may be a cause of failure for UK trained candidates and international medical graduates’*.^3^ Despite a judicial review in 2014^4^ and subsequent narrative review finding no evidence of racial discrimination,^5^ there has been ongoing focus from some commentators on addressing unconscious bias, changing assessments, or addressing other unproven factors such as self-efficacy, and inclusion and relationships with educators and peers.^6^

The Royal College of General Practitioners (RCGP), General Medical Council (GMC), and Health Education England (HEE) — responsible for licensing of GPs, medical regulation, and postgraduate education, respectively — in response to the judicial review, have undertaken initiatives related to assessment, training, and research, which are designed to address differential attainment.

These include the following: aligning the curriculum and assessments to GMC Excellence-by-design standards, which have fairness as a guiding principle; reviewing and revising assessments where possible to reflect the UK patient population and reduce potential for differential attainment, including stakeholder engagement, pilots, and equality impact assessments for new or revised assessments; recruiting examiners and exam advisers from underrepresented groups and providing mandatory equality, diversity, and inclusion training; developing educational events and resources to support trainers and candidates including those who have failed exams in exam preparation; reviewing results, reports, guidance, and feedback to minimise risk of unconscious bias and to meet accepted guidelines for those with disabilities; and finally prioritising research into differential attainment.^7^^–^^9^

Confounding factors implicated in differential attainment by doctors’ ethnic group include age, sex, and place of primary medical qualification; although these have been included in previous studies of differential attainment, other factors, also related to ethnic group, such as declared disability or performance at selection into GP training, have less often been accounted for.^10^^–^^12^ Complex educational and social factors may affect educational progress.^13^ The potential contribution of these structural inequalities^14^ is recognised in the term ‘awarding’ rather than ‘attainment’ gap.^15^ These factors are rarely included in statistical models because data are lacking.^13^ Performance at selection into specialty training, reflecting prior education and medical education, may affect endpoint attainment.^16^

**Table:** How this fits in

Differential attainment is widely found in undergraduate and postgraduate medical examinations. It has been suggested that subjective bias due to racial discrimination in clinical skills assessments may be a cause of examination failure for UK-trained ethnic minority candidates and international medical graduates. To the authors’ knowledge, no previous study has examined differential attainment in all components of GP licensing assessments, including the Workplace-Based Assessment, considering scores at selection in GP specialty training. Ethnic background did not reduce the chance of passing GP licensing tests once sex, place of primary medical qualification, declared disability, and selection (Multi-Specialty Recruitment Assessment [MSRA]) scores were considered. Doctors admitted to GP specialty training, who are in the lowest MSRA score bands, may need additional support during training to maximise their chances of achieving licensing, regardless of their ethnic group or other demographic characteristics.

Selection for general practice training has involved the following three-stage process:^17^

- stage 1, administrative: candidates provide proof of eligibility for UK specialty training, including proof of foundation-level competence;
- stage 2, Multi-Speciality Recruitment Assessment (MRSA): a computer-based multiple-choice question examination, including both clinical problem-solving items and Situational Judgement Tests, developed and delivered by the National Recruitment Office as a shortlisting tool for many medical specialties, including general practice; and
- stage 3, Selection Centre (SC): a GP-specific face-to-face assessment using objective structured clinical examination (OSCE)-style simulations and a written test for those scoring <575 on the MSRA. The SC was suspended in 2020 during the COVID-19 pandemic.

The MRCGP licensing test consists of the following three components: a computer-based Applied Knowledge Test (AKT); the Clinical Skills Assessment (CSA), a 13-station OSCE with role-players;^18^ and Workplace-Based Assessment (WPBA), which informs an Annual Review of Competence Progression (ARCP) panel. Since 2020, the CSA was replaced by the Recorded Consultation Assessment (RCA), which uses 13 audio or video recordings of real patient consultations carried out and selected by candidates. Candidates are allowed up to five attempts at the AKT and the CSA or RCA, which includes four standard attempts and an exceptional fifth attempt, which most who request it are allowed.

Previous research has found that scores at selection into GP training were predictive of performance in the AKT and CSA.^18^^,^^19^ No previous studies have explored differential attainment in WPBA–ARCP and evidence that differences in performance reflect prior academic performance is lacking.^6^

This study aimed to investigate the extent of differential attainment by ethnic group in all components of the MRCGP, including the AKT, CSA, and WPBA–ARCP, while considering important potential confounders such as performance at selection into GP training, sex, disability, and place of primary medical qualification.

## METHOD

### Design

A longitudinal design was employed, using retrospective data for doctors’ performance from selection to the end of GP training, linking selection, licensing, and demographic data from doctors entering GP specialty training in 2016. The research question was as follows: is performance in the MRCGP (AKT, CSA, RCA, or WPBA– ARCP) different in ethnic minority versus White doctors? The objective was to investigate differences in performance in the MRCGP comparing ethnic minority with White doctors. The null hypothesis was that there was no difference in performance between ethnic minority and White doctors.

### Setting, data collection, and processing

All doctors entering UK GP specialty training in 2016 were included. They were followed up with all licensing test outcomes until the end of 2021.

MSRA and SC scores (available only for those scoring <575 on the MSRA) for doctors undertaking selection tests in 2016 were linked with their AKT, CSA, RCA, and WPBA–ARCP outcomes to 2021.

Individual candidate data provided by the GP National Recruitment Office, HEE, were linked with assessment outcomes and demographic data at the RCGP and transferred securely as a pseudonymised dataset, under a data-sharing agreement with the research team.

Individual candidates were assigned a unique (non-personally identifiable) number to link the various assessments to demographic data, including sex, ethnic group, country of graduation, and declared disability (specific learning difficulties and other physical disabilities), and assessment results, including overall scores, scores for assessment subdomains, and outcomes of pass (1) or fail (0).

Ethnic group was divided into the following three categories: White, ethnic minority, and mixed. White included White British, White Irish, and any other White background. Ethnic minority included Asian (Bangladeshi, Indian, or Pakistani) or Asian British, Chinese, Black (African or Caribbean) or Black British, any other Asian background, any other Black background, and any other ethnic group. Mixed ethnicity included mixed White and Asian, mixed White and Black African, mixed White and Black Caribbean, and any other mixed background.

Reasonable adjustments were provided for candidates with disabilities depending on their needs and requirements, which were based on a specialist assessment for written examinations and clinical assessments, including extra time (the standard is 25% additional time), a separate room for testing, and extra breaks.^20^

Binary variables included the following: country of graduation (UK versus non-UK graduates), sex (male versus female), and declared disability (declared disability recorded versus no declaration of disability).

WPBAs are undertaken throughout the year and progress of the trainee is reviewed by a panel at their ARCP at the end of each academic year. Outcomes were categorised as ‘standard’ (for example, achieving progress and competencies at the expected rate or gaining all required competencies for completing training) or ‘developmental’ (for example, further development of specific competences required), and there is also the option of releasing the candidate from the training programme.

The main outcome variables were pass (1) or fail (0) for the AKT, CSA, or RCA examinations, and presence of only standard ARCP outcomes (1) versus at least one developmental outcome or release from training (0).

MSRA scores were divided into 12 score bands and SC scores were divided into seven score bands, which were based on distribution of data and to achieve bands narrow enough to precisely identify candidates with differing performance.

It was estimated that a minimum sample size of 830 would be needed to see even a small effect size of 0.02 with five predictors, power 90%, and probability 0.05.^21^

### Statistical analysis

Descriptive statistics were used, indicating percentages of candidates passing each assessment and mean scores for CSA and RCA subdomains. Multivariable logistic regression models were used to determine the effect of ethnic group on licensing performance once sex, country of primary medical qualification, declared disability, and MSRA score bands were accounted for. Assumptions of no multicollinearity and no outliers were checked. Odds ratios (ORs), representing the odds that the outcome would occur given a predictor, compared with the odds of the outcome occurring in the absence of that predictor (that is, at baseline), and pseudo R^2^, representing the certainty with which the model can predict the dichotomous outcome (y = 0 or y = 1), were reported.

## RESULTS

A total of 3429 GP trainees who took the MSRA in 2016 were included, of which 2883 took the AKT (Supplementary Figure S1), 2313 the CSA, and 545 the RCA (Supplementary Figure S2), and 3168 were graded on the WPBA–ARCP (Supplementary Figure S3). The doctors were of different sex (female 63.8% versus male 36.2%), ethnic group (White British 54.0%, minority ethnic 43.0%, or mixed 3.0%), country of primary medical qualification (UK 76.8% and non-UK 23.2%), and declared disability (disability declared 12.0% and no disability declared 88.0%), with 1633 (50.2%) White UK doctors, 122 (3.8%) White non-UK doctors, 861 (26.5%) ethnic minority UK doctors, and 637 (19.6%) ethnic minority non-UK doctors (*n* = 176 missing data) (Supplementary Table S1). There were no missing data for the variables of interest.

Disabilities declared were chiefly specific learning difficulties (86.3% of all disabilities), but also included physical disability (1.6%), visual impairment (1.6%), hearing impairment (1.2%), and other disabilities (9.3%) (data not shown).

Pass rates were the highest for AKT, with 98.2% of candidates passing within the study period, followed by the CSA (92.4%) and RCA (85.8%). Pass rates were lowest for the RCA alone, but the number of possible attempts was lowest (three compared with five for AKT and CSA) and circumstances were different owing to its introduction during the COVID-19 pandemic. Raw pass rates at the first attempt were generally higher for White compared with mixed and ethnic minority candidates for the AKT (86.9%, 86.6%, and 61.6%), CSA (80.3%, 87.1%, and 66.4%), or RCA (95.5%, 88.2%, and 77.9%) (Supplementary Table S2).

MSRA score bands were the strongest predictors for all GP licensing outcomes at the 5-year point (AKT, CSA, RCA, and WPBA–ARCP). Lower SC score bands corresponded to poorer GP training outcomes but adding SC scores did not change the predictive validity of the MSRA. Therefore, the SC did not add further information to MSRA scores and were therefore not included in the logistic regression models.

Pass rates in AKT, CSA, or RCA and standard outcomes in the ARCP for ethnic minority doctors were no longer significantly different for White British doctors when MRSA scores and demographic factors, including sex, country of qualification, and declared disability, were considered. Conversely, ethnic minority doctors did significantly better compared with White British doctors in the AKT (OR 2.05, 95% CI = 1.03 to 4.10, *P* = 0.042) once these factors were taken into account, as seen in Table 1. There were no significant differences on the other assessments: CSA (OR 0.72, 95% CI = 0.43 to 1.20, *P* = 0.201), RCA (OR 0.48, 95% CI = 0.18 to 1.32, *P* = 0.156), or WPBA–ARCP (OR 0.70, 95% CI = 0.49 to 1.01, *P* = 0.057) (as seen in Tables 2–4).

**Table 1.** Multivariable logistic regression model showing factors independently associated with passing the Applied Knowledge Test

**Predictor**	**OR (95% CI)**	**Standard error**	***P*-value**
**Sex**			
Female	1		
Male	1.29 (0.70 to 2.36)	0.40	0.411

**Ethnic group**			
White	1		
Ethnic minority	2.05 (1.03 to 4.10)	0.72	0.042
Mixed	1.20 (0.14 to 10.00)	1.30	0.865

**Qualification country**			
UK	1		
Non-UK	1.17 (0.54 to 2.54)	0.46	0.686

**Declared disability**			
No	1		
Yes	0.86 (0.42 to 1.77)	0.32	0.687

**MSRA bands**			
<400	1		
400–419	3.47 (1.28 to 9.36)	1.76	0.014
420–439	4.29 (1.42 to 12.94)	2.42	0.010
440–459	6.86 (2.40 to 19.11)	3.68	<0.001
460–479	9.93 (3.18 to 31.03)	5.77	<0.001
480–499	15.34 (4.35 to 54.08)	9.86	<0.001
500–519	37.53 (8.37 to 168.40)	28.75	<0.001
520–539	53.30 (9.58 to 296.52)	46.67	<0.001
540–559	104.06 (11.28 to 959.69)	117.95	<0.001

**Constant**	1.69 (0.55 to 5.21)	0.97	<0.001

*Pseudo R^2^ = 0.13,* χ*^2^(13) = 56.78,* P*<0.001. Bands 10, 11, and 12 not included in the model because they perfectly predict passing the Applied Knowledge Test. MSRA = Multi-Specialty Recruitment Assessment. OR = odds ratio.*

**Table 2.** Multivariable logistic regression model showing factors independently associated with passing the Clinical Skills Assessment

**Predictor**	**OR (95% CI)**	**Standard error**	***P*-value**
**Sex**			
Female	1		
Male	0.58 (0.39 to 0.86)	0.12	0.007

**Ethnic group**			
White	1		
Ethnic minority	0.72 (0.43 to 1.20)	0.19	0.201

**Qualification country**			
UK	1		
Non-UK	0.27 (0.16 to 0.45)	0.07	<0.001

**Declared disability**			
No	1		
Yes	0.38 (0.24 to 0.61)	0.09	<0.001

**MSRA bands**			
<400	1		
400–419	0.92 (0.40 to 2.10)	0.39	0.848
420–439	2.58 (0.97 to 6.88)	1.29	0.059
440–459	1.04 (0.47 to 2.33)	0.43	0.915
460–479	0.99 (0.44 to 2.22)	0.41	0.972
480–499	1.48 (0.61 to 3.60)	0.67	0.389
500–519	4.00 (1.31 to 12.23)	2.28	0.015
520–539	2.47 (0.85 to 7.15)	1.34	0.097
560–579	11.58 (1.36 to 98.83)	12.67	0.025
580–599	6.86 (0.80 to 58.98)	7.53	0.080

**Constant**	17.76 (6.83 to 46.20)	8.66	<0.001

*Pseudo R^2^ = 0.21,* χ*^2^(13) = 178.87, P<0.001. Bands 9 and 12 and mixed ethnicity were not included in the model because they perfectly predict passing the Clinical Skills Assessment. MSRA = Multi-Specialty Recruitment Assessment. OR = odds ratio.*

**Table 3.** Multivariable logistic regression model showing factors independently associated with passing the Recorded Consultation Assessment

**Predictor**	**OR (95% CI)**	**Standard error**	***P*-value**
**Sex**			
Female	1		
Male	0.74 (0.37 to 1.45)	0.25	0.377

**Ethnic group**			
White	1		
Ethnic minority	0.48 (0.18 to 1.32)	0.25	0.156
Mixed	0.14 (0.20 to 0.94)	0.13	0.043

**Qualification country**			
UK	1		
Non-UK	0.30 (0.11 to 0.80)	0.15	0.017

**Declared disability**			
No	1		
Yes	0.58 (0.27 to 1.23)	0.22	0.156

**MSRA bands**			
<400	1		
400–419	5.46 (1.61 to 18.51)	3.40	0.006
420–439	5.98 (1.27 to 28.18)	4.73	0.024
440–459	5.00 (1.50 to 16.65)	3.07	0.009
460–479	2.60 (0.81 to 8.24)	1.53	0.107
480–499	6.24 (1.50 to 25.95)	4.54	0.012
500–519	5.95 (1.16 to 30.47)	4.96	0.032
520–539	9.89 (0.89 to 109.88)	12.15	0.062
560–579	9.97 (0.71 to 142.06)	13.52	0.090
580–599	8.03 (0.67 to 95.92)	10.16	0.100

**Constant**	7.69 (1.55 to 38.28)	6.30	0.013

*Pseudo R^2^ = 0.18, X^2^(14) = 54.75,* P*<0.001. Bands 9 and 12 were not included in the model because they perfectly predict passing the Recorded Consultation Assessment. MSRA = Multi-Specialty Recruitment Assessment. OR = odds ratio.*

**Table 4.** Multivariable logistic regression model showing factors independently associated with only standard WPBA–ARCP outcomes

**Predictor**	**Odds ratio (OR)**	**Standard error**	***P*-value**
**Sex**			
Female	1		
Male	0.50 (0.37 to 0.68)	0.08	<0.001

**Ethnic group**			
White	1		
Ethnic minority	0.70 (0.49 to 1.01)	0.13	0.057
Mixed	0.62 (0.26 to 1.46)	0.27	0.274

**Qualification country**			
UK	1		
Non-UK	0.50 (0.34 to 0.74)	0.10	0.001

**Declared disability**			
No	1		
Yes	0.33 (0.23 to 0.49)	0.07	<0.001

**MSRA bands**			
<400	1		
400–419	0.81 (0.34 to 1.94)	0.36	0.639
420–439	1.14 (0.47 to 2.76)	0.51	0.775
440–459	1.14 (0.50 to 2.61)	0.48	0.754
460–479	1.56 (0.67 to 3.60)	0.67	0.303
480–499	1.58 (0.68 to 3.69)	0.68	0.292
500–519	1.72 (0.72 to 4.07)	0.76	0.217
520–539	4.18 (1.59 to 11.01)	2.07	0.004
540–559	3.30 (1.24 to 8.83)	1.66	0.017
560–579	3.32 (1.20 to 9.21)	1.73	0.021
580–599	12.06 (2.39 to 60.87)	9.96	0.003
≥600	5.65 (1.10 to 29.06)	4.72	0.038

**Constant**	8.04 (3.39 to 19.05)	3.54	<0.001

*Pseudo R^2^ = 0.23, X^2^(16) = 455.88,* P*<0.001. ARCP = Annual Review of Competency Progression. MSRA = Multi- Specialty Recruitment Assessment. OR = odds ratio.*

Sex differences in performance were apparent in the CSA and WPBA–ARCP, with males doing significantly worse than females (Tables 2 and 4). International medical graduates (IMGs) performed significantly less well than UK-trained graduates in the CSA, RCA, and ARCP but not the AKT (Tables 1–4). Finally, candidates who declared a disability, most of whom stated they had a specific learning difficulty, performed significantly less well in the CSA and ARCP but not the AKT or RCA, although numbers included were small, particularly in the RCA.

**Figure 1.**
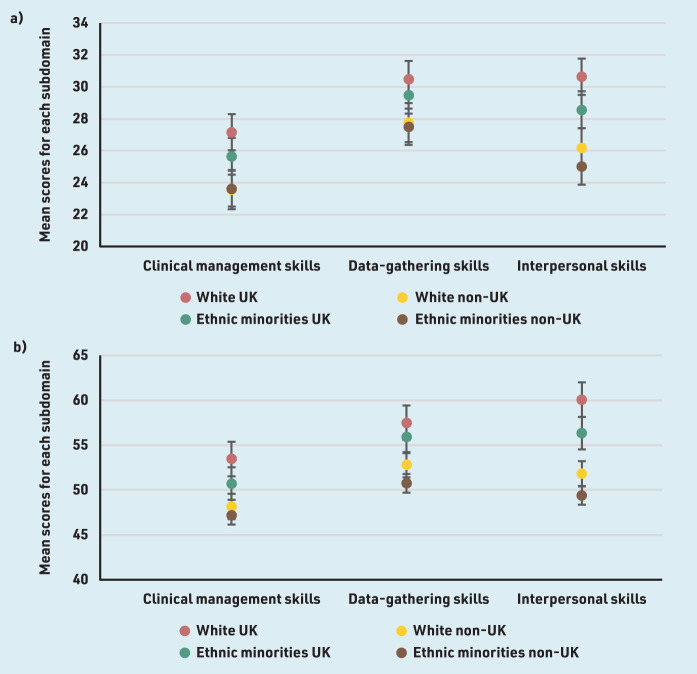
*Performance as indicated by mean scores for all subdomains of a) the Clinical Skills Assessment and b) Recorded Consultation Assessment.*

White or ethnic minority IMGs had lower pass rates more pronounced in the CSA and in-training ARCP outcomes than UK doctors (Supplementary Figure S4). Logistic regression models accounting for sex, disability, and prior MSRA attainment with White UK doctors as comparators indicated that overseas-trained ethnic minority doctors performed significantly better on the AKT (OR 2.52, 95% CI = 1.03 to 6.16, *P* = 0.043) (Table 5). Both White (OR 0.19, 95% CI = 0.07 to 0.48, *P* = 0.001) and ethnic minority (OR 0.15, 95% CI = 0.08 to 0.30, *P*<0.001) doctors not graduating in the UK performed significantly less well on the CSA, but this was not the case for ethnic minority doctors graduating in the UK (OR 0.55, 95% CI = 0.28 to 1.09, *P* = 0.086). Only ethnic minority non-UK doctors performed significantly less well on the RCA (OR 0.11, 95% CI = 0.03 to 0.45, *P* = 0.002). Being a White (OR 0.34, 95% CI = 0.18 to 0.62, *P*<0.001) or ethnic minority (OR 0.29, 95% CI = 0.19 to 0.43, *P<*0.001) IMG predicted a significantly lower likelihood of obtaining only standard ARCP outcomes, but this was not the case for ethnic minority UK graduates (OR 0.70, 95% CI = 0.49 to 1.01, *P* = 0.055). Detailed results can be seen in Table 5. All other groups had a poorer performance on all subdomains of the CSA and RCA compared with White UK graduates, but this was more pronounced in White and ethnic minority IMGs on the interpersonal skills subdomain (Figure 1).

**Table 5.** Predictors of pass rates for all licencing tests and for presence of only standard in-training outcomes

**Predictors**	**AKT**		**CSA**		**RCA**		**WPBA—ARCP**	

	**OR (95% CI)**	***P*-value**	**OR (95% CI)**	***P*-value**	**OR (95% CI)**	***P*-value**	**OR (95% CI)**	***P*-value**
**Ethnic group and country of PMQ**								
White UK PMQ	1		1		1		1	
White non-UK PMQ	2.08 (0.82 to 5.26)	0.496	0.19 (0.07 to 0.48)	0.001	0.26 (0.04 to 1.49)	0.129	0.34 (0.18 to 0.62)	<0.001
Ethnic minority UK PMQ	2.07 (0.82 to 5.26)	0.123	0.55 (0.28 to 1.09)	0.086	0.31 (0.07 to 1.31)	0.111	0.70 (0.49 to 1.01)	0.055
Ethnic minority non-UK PMQ	2.52 (1.03 to 6.16)	0.043	0.15 (0.08 to 0.30)	<0.001	0.11 (0.03 to 0.45)	0.002	0.29 (0.19 to 0.43)	<0.001

**Sex**								
Female	1		1		1		1	
Male	1.25 (0.69 to 2.30)	0.462	0.56 (0.38 to 0.83)	0.004	0.71 (0.37 to 1.35)	0.294	0.45 (0.35 to 0.58)	<0.001

**Declared disability**								
No	1		1		1		1	
Yes	0.88 (0.43 to 1.81)	0.722	0.38 (0.24 to 0.60)	<0.001	0.56 (0.27 to 1.17)	0.124	0.29 (0.21 to 0.41)	<0.001

**Prior attainment**								
MSRA scores	1.03 (1.02 to 1.04)	<0.001	1.01 (1.00 to 1.01)	<0.001	1.01 (1.00 to 1.02)	0.137	1.01 (1.01 to 1.02)	<0.001

*After accounting for AKT scores for the CSA outcome, non-UK ethnic minorities remained non-significant (OR 0.62, 95% CI = 0.31 to 1.22,*P*= 0.168) and the other two ethnic categories significant: White non-UK (OR 0.21, 95% CI = 0.08 to 0.55,* P*= 0.001) and ethnic minority non-UK (OR 0.15, 95% CI = 0.08 to 0.35,* P*<0.001). AKT = Applied Knowledge Test. CSA = Clinical Skills Assessment. MSRA = Multi-Specialty Recruitment Assessment. OR = odds ratio. PMQ = primary medical qualification. RCA = Recorded Consultation Assessment. WPBA—ARCP = Workplace-Based Assessment and Annual Review of Competency Progression.*

## DISCUSSION

### Summary

Ethnic minority doctors performed no worse in GP licensing assessments when MSRA scores and demographic factors (sex, country of qualification, and declared disability) were considered. Ethnic minority doctors in general (OR 2.05, 95% CI = 1.03 to 4.10, *P* = 0.042) and non-UK ethnic minority doctors in particular (OR 2.52, 95% CI = 1.03 to 6.16, *P* = 0.043) were significantly more likely to pass the AKT compared with White British doctors once these factors were taken into account.

### Strengths and limitations

There were high rates of completeness for outcome and demographic data. This study followed the 2016 cohort to 2021 as it was anticipated that most participants undergoing ‘standard’ 3-year full-time or extended GP training programmes would by then have attempted licensing assessments. However, not all participants who were unsuccessful in licensing tests would have had the opportunity to take them the permitted four times. For AKT and CSA this number was small (only 6% of candidates), but it involved all participants for the RCA who could only attempt this assessment three times by the end of the study.

Candidates on training extensions, maternity leave, and so on may have successfully completed training after the study end. The absence of significant differences for IMGs and those with declared disabilities in the RCA may have been owing to the smaller numbers of candidates who were able to take this assessment.

The analysis simplified categories of doctors who had qualified in the UK or overseas, those from ethnic minorities, or those with disabilities together, which does not take into account differences by medical school, country of primary qualification, ethnic group, or nature of disability. This was partly because the study did not have data on subcategories but also because increasing the number of categories would have provided groups that were too small for analysis.

### Comparison with existing literature

Previous studies of differential attainment have included sex, place of primary medical qualification, and declared disability as covariates,^10^^,^^11^ but selection scores, although known to predict licensing outcomes, have rarely been included in analyses.^18^ In this study, MSRA was one of the main factors influencing outcomes, with ethnic group ceasing to be a significant predictor for any endpoint assessment when MSRA scores were taken into account.

A study examining the predictive value of selection tests showed strong correlations with educational supervisor rating at 1 year and performance in the AKT and CSA.^18^ Another study combining MSRA and SC scores to investigate prediction of performance in AKT and CSA in one deanery found good prediction for the combined score.^19^

In the present study, IMGs were just as likely to pass AKT but less likely to pass CSA or RCA or achieve only standard ARCP outcomes once MSRA scores and other demographic factors were accounted for. The explanations for differential attainment in IMGs are complex and multiple but are likely owing to ‘*difference in training experience and other cultural factors between candidates trained in the UK and abroad’*.^3^ These may include differences at recruitment to medical school or postgraduate training, during training and performance at assessments, cultural barriers (language difficulties, lack of understanding of cultural norms, and bias against seeking support or additional training), more limited professional networks (lack of mentorship or peer support), social challenges (poor work–life balance, separation from family, and lack of social support outside the work setting), and psychological difficulties (stress, anxiety, and burnout).^13^^,^^22^

Another factor affecting performance of non-UK graduates in clinical licensing tests may be differences in initial medical training, where a doctor-centred rather than patient-centred approach to consulting may be taught and learnt.^23^ These results suggest that prior attainment and training experience are the main factors driving the successful performance on the various licensing assessments.

Overall, the findings indicate that prior attainment and a primary medical qualification outside the UK were the main factors influencing performance on licensing assessments. A previous study examining CSA performance in ethnic minority doctors graduating in the UK or overseas indicated that IMGs had the poorest performance.^3^ The present study showed that prior attainment as recorded by MSRA scores, having a disability, being male, and graduating outside of the UK as a White or ethnic minority person were all significant predictors of lower pass rates on the CSA, but being of ethnic minority background and graduating in the UK was not. It is extremely unlikely that all these findings are due to subjective bias.

A more plausible interpretation would be that the CSA assesses certain skills that pose more difficulties for certain candidates including males, those with a declared disability, and those of different ethnic backgrounds graduating outside the UK. Moreover, the previous study did not compare performance directly, but ran independent logistic regression models and considered the differences in likelihood of failing the CSA.^3^ The present study used the White UK graduate category as baseline and directly compared performance with all other ethnic groups on all licensing assessments. Importantly, the MSRA scores were used as an indicator of prior attainment, rather than AKT scores, which form part of the licensing assessments, with some candidates taking the CSA before the AKT. In the analysis, even accounting for AKT scores, the ethnic minority British group did not have a significantly poorer performance on the CSA.

Lastly, it is important to consider that the licensing assessments are based on a well-established pedagogy, and they are internationally recognised and used, which may indicate that candidates who fail are simply not ready for independent general practice.

## Implications for research and practice

Differential attainment is present throughout the educational and training journey, and the correlation across longitudinal assessments, termed the academic backbone by McManus and colleagues,^16^ is also seen in selection and licensing assessments.

The finding that ethnic status had no significant effect on performance at licensing assessments once selection scores and other demographic factors were accounted for suggests that, rather than the explanation being related to ethnic group, the reason for these differences, at least at licensing, is owing to differences on entering GP training rather than examiner bias, poorer relationships with educators and peers, or environment.^24^

GP trainees should receive educational support appropriate to their needs, whatever their ethnic group or other demographic characteristics, particularly doctors admitted to training with low selection scores who may need additional support to maximise their chances of successful licensing. The present findings do not conflict with evidence that differential attainment by ethnic group, and potential factors associated with it, may be operating at medical school^15^ or even earlier in the educational journey.

A previous systematic review, suggesting areas for support for doctors with protected characteristics, identified several factors that could influence differential attainment including learning and working environment, training experience and progression, learning and knowledge, and behavioural factors such as motivation and affect.^25^ Interventions aimed at addressing differential attainment should consider these factors, but more rigorous research is needed to investigate the effect of possible interventions to address underperformance.

Educational interventions focusing on candidates who fail one component of the assessment, although these may be helpful,^26^ could be replaced by support offered at the outset of training, for example, ensuring fairness in allocation of more sought after training practices and rotations, and enhanced educational provision, such as the Scottish Trainee Enhanced Programme (STEP).^27^ This should be available to those who have been found to have low scores at selection and others who feel they may benefit, for example, IMGs, although this will need to be done carefully and communicated sensitively to avoid stigmatising this group of trainees.

More robust intervention development and stronger evaluation designs would add to the quality of evidence. Future studies should use larger datasets to explore differences by medical school, country of primary qualification, ethnic group, or nature of disability in greater detail and other factors that contribute to variation in performance at entry into specialty training for general practice. In addition, further studies should explore the relationship of entry standards to licensing outcomes and the factors that add value during training and improve subsequent performance.

In conclusion, ethnic background did not reduce the chance of passing GP licensing tests once sex, place of primary medical qualification, declared disability, and MSRA scores were considered. Comparing candidate scores by ethnic group creates a false impression of differential attainment, which should be addressed by routinely taking these other factors into account. Doctors admitted to GP specialty training in the lowest MSRA score bands may need additional support during training to maximise their chances of achieving licensing, regardless of their ethnic group or other demographic characteristics.

## Data

Data will be available from the corresponding author on reasonable request with appropriate ethics and governance approvals.

## References

[1] Wakeford R, Farooqi A, Rashid A, Southgate L (1992). Does the MRCGP examination discriminate against Asian doctors?. BMJ.

[2] Rendel S, Foreman P, Freeman A (2015). Licensing exams and judicial review: the closing of one door and opening of others?. Br J Gen Pract.

[3] Esmail A, Roberts C (2013). Academic performance of ethnic minority candidates and discrimination in the MRCGP examinations between 2010 and 2012: analysis of data. BMJ.

[4] Royal Courts of Justice (2014). R (on the application of BAPIO Action Limited) v Royal College of General Practitioners and another EWHC 1416.

[5] Regan de Bere S, Nunn S, Nasser M (2015). Understanding differential attainment across medical training pathways: a rapid review of the literature.

[6] Shah R, Ahluwalia S (2019). The challenges of understanding differential attainment in postgraduate medical education. Br J Gen Pract.

[7] Withnall R MRCGP annual report covering 2020/21.

[8] General Medical Council (2017). *Promoting excellence — equality and diversity considerations*.

[9] Health Education England Differential attainment: addressing differential attainment in primary care. https://www.hee.nhs.uk/our-work/equality-diversity-inclusion/differential-attainment.

[10] Asghar Z, Williams N, Denney M, Siriwardena AN (2019). Performance in candidates declaring versus those not declaring dyslexia in a licensing clinical examination. Med Educ.

[11] Asghar ZB, Siriwardena AN, Elfes C (2018). Performance of candidates disclosing dyslexia with other candidates in a UK medical licensing examination: cross-sectional study. Postgrad Med J.

[12] Botan V, Laparidou D, Phung VH (2022). Candidate perceptions of the UK Recorded Consultation Assessment: cross-sectional data linkage study. Educ Prim Care.

[13] Woolf K, Rich A, Viney R (2016). Fair training pathways for all: understanding experiences of progression Final report.

[14] Teherani A, Hauer KE, Fernandez A (2018). How small differences in assessed clinical performance amplify to large differences in grades and awards: a cascade with serious consequences for students underrepresented in medicine.. Acad Med.

[15] Fyfe M, Horsburgh J, Blitz J (2022). The do’s, don’ts and don’t knows of redressing differential attainment related to race/ethnicity in medical schools. Perspect Med Educ.

[16] McManus IC, Woolf K, Dacre J (2013). The Academic Backbone: longitudinal continuities in educational achievement from secondary school and medical school to MRCP(UK) and the specialist register in UK medical students and doctors. BMC Med.

[17] Plint S, Patterson F (2010). Identifying critical success factors for designing selection processes into postgraduate specialty training: the case of UK general practice. Postgrad Med J.

[18] Patterson F, Lievens F, Kerrin M (2013). The predictive validity of selection for entry into postgraduate training in general practice: evidence from three longitudinal studies. Br J Gen Pract.

[19] Wakeford R (2012). International medical graduates’ relative under-performance in the MRCGP AKT and CSA examinations. Educ Prim Care.

[20] Foreman P (2018). Managing access arrangements for candidates requesting adjustments in high stakes assessments.

[21] Faul F, Erdfelder E, Buchner A, Lang AG (2009). Statistical power analyses using G*Power 3.1: tests for correlation and regression analyses. Behav Res Methods.

[22] Pattinson J, Blow C, Sinha B, Siriwardena A (2019). Exploring reasons for differences in performance between UK and international medical graduates in the Membership of the Royal College of General Practitioners Applied Knowledge Test: a cognitive interview study. BMJ Open.

[23] Jalal M, Bardhan KD, Sanders D, Illing J (2019). International: overseas doctors of the NHS: migration, transition, challenges and towards resolution. Future Healthc J.

[24] Woolf K, Viney R, Rich A (2018). Organisational perspectives on addressing differential attainment in postgraduate medical education: a qualitative study in the UK. BMJ Open.

[25] Work Psychology Group (2018). Evaluating the impact of interventions aimed at addressing variation in progression associated with protected characteristics known as ‘differential attainment’.

[26] Hawkridge A, Molyneux D (2019). A description and evaluation of an educational programme for North West England GP trainees who have multiple fails in the Clinical Skills Assessment (CSA). Educ Prim Care.

[27] NHS Education for Scotland Trainee information. https://www.scotlanddeanery.nhs.scot/trainee-information/gp-specialty-training/scottish-trainee-enhanced-programme-step.

